# Supplementary material for: Single-pixel infrared imaging thermometry maps human inner canthi temperature
Source: Nat Commun. 2025 Oct 6;16:8885. doi: 10.1038/s41467-025-64125-3 (PMC12501256; doi:10.1038/s41467-025-64125-3)
Supplement: Supplementary file 1 — Supplementary Information [file 41467_2025_64125_MOESM1_ESM.pdf]

SUPPLEMENTARY INFORMATION

Single-pixel infrared imaging thermometry maps human inner canthi temperature

Cheng Jiang[†,1], Patrick Kilcullen[†,1], Yingming Lai[1], Tsuneyuki Ozaki[1], and Jinyang Liang[*,1]

[1]Centre Énergie Matériaux Télécommunications, Institut National de la Recherche Scientifique, Université du Québec, 1650 boulevard Lionel-Boulet, Varennes, Québec J3X1P7, CANADA

[†]These authors contributed equally to this work

[*]Corresponding author: jinyang.liang@inrs.ca

Supplementary Note 1: Details of alignment between the mask plate and window plate

In single-pixel infrared imaging thermometry (SPIRIT), a window plate and a mask plate are used to encode thermal radiation. As shown in Supplementary Figs. 1a and b, the mask plate and window plate are fabricated on 2” round plates via laser drilling. A close-up view of Supplementary Fig. 1a reveals that each pixel, designed to be 250 µm × 250 µm, has a 200-µm width and a 50-µm gap to avoid the “island pixel” issue.

As the reconstructed image quality largely depends on the encoding accuracy, it was necessary to meticulously align the mask plate and window plate. The first step was to check whether the mask’s scanning motion was perpendicular to the encoding beam path. This examination was conducted by checking the two vertical alignment markers with the same horizontal coordinate (marked by the yellow arrows in Supplementary Fig. 1a). A thermal camera (M384D, Yoseen Infrared) was used to measure the horizontal positions of the alignment markers, which subsequently guided the mask plate’s rotation until the two measured horizontal positions were equal.

The second step was to align the window plate with the mask plate. The window plate was fixed to a rotational mount attached to an x-z translation stage. The window plate was cautiously moved towards the mask plate until they almost touched. The thermal camera continuously monitored the encoded thermal radiation transmitted through the window. Shown in Supplementary Figs. 1c–f, the exact one-to-one correspondence between the encoding masks (Supplementary Figs. 1c and e) and the images acquired by the thermal camera (Supplementary Figs. 1d and f) confirms the accurate alignment between the window plate and mask plate. Similarly, a systematic check of all 95 masks (i.e., 91 encoding masks and 4 run-way masks) was performed to verify the accurate alignment of each encoding mask.

Supplementary Note 2: System synchronization and data acquisition

SPIRIT's measurement procedure is illustrated in Supplementary Fig. 2a. It involves vertical scanning of the mask plate by a motorized translation stage in stepper mode and the signal collection via a HgCdTe photodiode in conjunction with an optical chopper and a lock-in amplifier. The optical chopper modulates the signals with a constant frequency. This modulated signal, along with the reference frequency, is sent to the lock-in amplifier for filtering and amplification. The signal is then digitized by a digitizer and finally stored as waveforms with a sampling rate of 20 MHz.

Illustrated in Supplementary Fig. 2b, the stored waveform is further processed by computing the bucket signals [i.e., y in Eq. (1)]. In particular, the waveform is registered with the motorized translation stage through pre-calibrated position signals associated with individual encoding masks. During vertical scanning, the motorized translation stage initially moves the mask plate upward until the first run-way mask is positioned 5 mm above the window. At this moment, the window is completely obstructed, corresponding to a low amplitude in the lock-in amplifier signal. When the scan starts, the signal gradually ramps to a peak value, corresponding to the transmitted thermal signal encoded by the first run-way mask of the mask (marked in Supplementary Fig. 2b). When the mask plate is scanned to the positions of the encoding masks, the motorized translation stage's position readout aligns with the pre-calibrated position. This match triggers the beginning of averaging the lock-in amplifier signals for 1 ms. Subsequently, this averaged result is stored as the bucket signal for the corresponding encoding mask.

Supplementary Note 3: Details of field-of-view co-registration

The co-registration of the FOVs of the single-pixel imaging setup and the visible camera is pivotal to SPIRIT's capacity to accurately image the human inner canthi. The frame sizes of SPIRIT are 11 × 7 pixels and 11 × 6 pixels for the left and right inner canthi, respectively, corresponding to the two regions in the FOV of 11 mm × 7 mm and 11 mm × 6 mm. Besides precise positioning, the system also needs to accommodate the anatomical variations across different individuals.

We used a human face mask, affixed to a chinrest and facing the SPIRIT system, to mimic the conditions of human experiments (Supplementary Fig. 3a). Two Peltier thermoelectric coolers, each with 8.8 mm × 8.8 mm in size, were adhered to the inner canthus regions on the face mask

and positioned 30 mm apart. The hot side of the Peltier cooler had a temperature of approximately 70°C, generating a substantial thermal contrast relative to the ambient temperature (i.e., 22°C). The visible camera in the SPIRIT system, mounted atop the HgCdTe photodiode, captured color images of the face mask to aid the co-registration.

Supplementary Figs. 3b–d show the operation of FOV co-registration. SPIRIT's reconstructions were compared with visible images. Then, the face mask was adjusted iteratively until the two results were matched. Finally, we recorded the pixel coordinates of both regions in the visible image and made digital boxes serving as guides for volunteers to accurately position their heads on the chinrest.

Supplementary Note 4: Implementation of lock-in amplification for SPIRIT

We verified the boost of signal-to-noise ratio (SNR) in SPIRIT measurements by using the lock-in amplifier. In the experiment, thermoelectric cooling was applied to the HgCdTe photodiode. A blackbody radiator, which maintained a stable and uniform temperature of 37.0 °C, was used as the object. We used a thick metal plate to first block the object from the imaging system and then quickly removed it. The first trial using only the photodiode led to a low SNR of 0.07, as indicated by the blue curve in Supplementary Fig. 4a.

Next, the lock-in amplifier was used to enhance the SNR. For SPIRIT, the optical chopper, operating at 850 Hz, was positioned in front of the HgCdTe photodiode to attach the carrier wave for lock-in detection. The time constant and low-pass filter level were tuned empirically to be 30 ms and 12 dB, respectively, to balance the noise reduction and the response time. As illustrated by the orange curve in Supplementary Fig. 4a, this method enhanced the SNR to 6.14. The importance of SNR to image reconstruction is validated by a comparison using both methods (Supplementary Fig. 4b).

Supplementary Note 5: Details of design search for construction of the encoded stripes

For SPIRIT's mask design, it was necessary to select n, the order of a constructible cyclic S-matrix, and m, the number of encoding patterns to be aggregated into the encoded stripe. To make the best possible selection, we carried out a comprehensive search of possible values of n and m that suited

the constraints of manufacturability and overall mask size while still providing a sufficient pixel count.

Although a wide variety of techniques can generate S-matrices, only three construction techniques are currently known for cyclic S-matrices [1], corresponding to values of n of the form (i) $n = p$ for prime p of the form $4w - 1$ for some positive integer w, (ii) $n = 2^w - 1$ for some positive integer w, and (iii) $n = pq$ for primes p and $q = p + 2$. First, to accommodate encoding patterns with rectangular shapes, n was required to possess a non-trivial factorization $n = ab$ with $a, b > 1$, thus eliminating constructions of the form (i) and some particular constructions of the form (ii) (e.g., the Mersenne primes). Taking a and b to be the height and width respectively of the system's 2D encoding patterns, the condition $1/2 < a/b < 2$ for some factorization of n was used to constrain the FOV's aspect ratio. This condition, while always met by constructions of the form (iii), provided a useful filter for cases of the form (ii) constructions with highly composite n.

For a given n with a factorization $n = ab$ specifying a possible encoding pattern design, two further pieces of information were considered. First, it is desirable that diagonal scanning ultimately results in the deployment of all encoding patterns contained within the measurement matrix. Given that row-major ordering is used to reshape rows of the measurement matrix into the encoding patterns of size $a \times b$, as Fig. 2 illustrates, a diagonal scan will deploy encoding patterns in the order $x(b + 1)$ with $x \in \{0, \dots, n - 1\}$ when considered modulo n. Consequently, it can be shown that the deployment of all encoding patterns under diagonal scanning occurs if and only if n and $b + 1$ are relatively prime [i.e., $\gcd(b + 1, n) = 1$]. Second, it is desirable that a given design allows for a truncation $m < n$ in the number of encoding patterns deployed by a physical mask while still producing a relatively uniform distribution of acquired data for well-conditioned 2D interpolation as part of image reconstruction. Mathematically, the distribution of data in the $a \times b$ array used for interpolation can be described for a given m as the set of row and column coordinates

$$\left\{ \left(\operatorname{mod}\left(\left\lfloor \frac{w(b+1)}{b} \right\rfloor, a \right),\ \operatorname{mod}(w(b+1), b) \right) \,\middle|\, w = 0, \dots, m-1 \right\}, \tag{S1}$$

where $\operatorname{mod}(\cdot, \cdot)$ denotes the modulo operation. As m increases, the $a \times b$ array is gradually filled by a set of diagonal stripes that wrap around the top/bottom and left/right edges. In searching for values of m producing distributions of points with some uniformity, it is reasonable to restrict consideration to values of m that produce an equal number of points r in each column of the array

(i.e., $m = rb$). In this case, the set of points occupying the first column produced by Eq. (S1) provides sufficient information to evaluate the evenness of the diagonal filling. This may be quantified by considering the set of values

$$\{\mathrm{mod}(t(b+1), a) \mid t = 0 \dots r-1\}\,, \tag{S2}$$

which consists of r distinct values. Writing these elements as a sequence $\tilde{a}_0, \tilde{a}_1, \dots, \tilde{a}_{r-1}$ arranged in strictly ascending order, the largest cyclic "gap" admitted by this distribution may be quantified as

$$d_r = \max\{\tilde{a}_1 - \tilde{a}_0, \tilde{a}_2 - \tilde{a}_1, \dots, \tilde{a}_{r-1} - \tilde{a}_{r-2}, \tilde{a}_0 + a - \tilde{a}_{r-1}\}\,, \tag{S3}$$

where the meaning of the last term, $\tilde{a}_0 + a - \tilde{a}_{r-1}$, is to anticipate the top/bottom wrapping effect of Laplace interpolation. The possible values of d_r can be shown to satisfy the lower bound $\lceil {}^{a}/_{r} \rceil \le d_r$. Thus, we may identify the values of m producing ideal distributions of data to be of the form $m = b\tilde{r}$ for values of $\tilde{r}$ equaling the lower gap bound $d_{\tilde{r}} = \lceil {}^{a}/_{\tilde{r}} \rceil$. Finally, in cases where multiple values of r produce equivalent values of d_r, only the smallest values of r are retained.

Various physical constraints determined an upper bound on the values of m compatible with the SPIRIT system. First, the 150-μm thickness of the metal mask substrate dictated the lower bound on the size of pixel apertures that could be both fabricated with acceptable accuracy via laser drilling and compatible with large numerical apertures. Second, the use of 2" diameter rotational mounts limited the total amount of space available for encoding mask fabrication to a disk approximately 1.75" in diameter, taking into account various space tolerances for clear aperture and encoding path layout. Together, these constraints dictated a rough theoretical limit of 300 for the number of encoding patterns that can be accommodated by SPIRIT's physical mask. Additionally, based on existing standards for imaging thermography, the requirement of a minimum of 3×3 pixels for the frame size [2] contributed by the encoding paths to each inner canthi imposed a lower resolution bound of $n = 18$.

Based on the above considerations, values of n in the range [18, 300] were searched for designs compatible with the construction of cyclic S-matrices and the various constraints on the reshaping dimensions a and b. The results of this search are compiled in Supplementary Table 1. Factors that influenced the final design selection included the desire for additional space allowances, high pixel count, and a moderate level of compression ratio m/n. Based on these preferences, the values chosen for the construction of the masks used in SPIRIT used a value of $n = 143$ corresponding to construction form (iii) with $a = 11$ and $b = 13$. This design admitted

a truncation length of $m = 91$, corresponding to a uniform distribution of data with a sampling ratio of 63.6%. MATLAB codes used for the implementation of the design search yielding the data of Supplementary Table 1 are included in Supplementary Software Package.

Supplementary Note 6: Step-by-step illustration of the generation of SPIRIT encoding patterns

The first step is to generate the first row of the cyclic S-matrix with $n = 11 \times 13 = 143$ (Supplementary Fig. 5a) using Eqs. (5)–(7) in Main Text. Then, this row is reshaped into a 2D pattern, as shown in Supplementary Fig. 5b. Next, four replicas of this 2D pattern are deliberately arranged in the way depicted in Supplementary Fig. 5c and Fig. 2b in Main Text. Finally, this block is duplicated and extended (Supplementary Fig. 5d). The first encoding pattern is marked by the red dashed box in Supplementary Fig. 5d. The diagonal scanning, whose direction is indicated by the red arrow, creates subsequent encoding patterns. According to the design search findings described in Supplementary Note 5, a total of $m = 91$ encoding patterns are produced in this manner.

A comparison between the distribution of bucket signals from a conventional linear scan (whose first encoding pattern and direction are illustrated by the cyan dashed box and the cyan arrow in Supplementary Fig. 5d) and that from SPIRIT in their 2D-reshaped bucket signals reveals considerable differences. As illustrated in Supplementary Fig. 5e, the conventional linear scan fills out the 1st to the 91st measurements while leaving the remaining areas empty. In contrast, SPIRIT's diagonal scan allows for a more uniform distribution of measurements across the 2D-reshaped bucket signals. The reduced maximum distances between unsampled and sampled signals confirm a substantial decrease in the gap of compressed measurements with SPIRIT (Fig. 2c), enhancing the quality of data interpolation and further image reconstruction.

Following the identification of the aggregated encoding patterns, four additional steps are taken to rearrange each pattern. First, as shown in Supplementary Fig. 5f, each encoding pattern is rotated by 45 degrees to facilitate vertical aggregation of subsequent patterns. To simultaneously image the inner canthi of human subjects, the pattern is divided into two sub-regions of 11×6 and 11×7 pixels in size, respectively. To account for the variable intercanthal distances and to maintain compatibility with the aggregation method, the left sub-region is horizontally flipped. Finally, in

accordance with SPIRIT’s imaging conditions, the entire pattern is rotated by 180 degrees to align with the desired FOV shape. Following this process, subsequent encoding patterns are aggregated to construct two encoding stripes, which were fabricated on the mask plate. An illustrative animation is shown in Supplementary Movie 1.

Supplementary Note 7: Simulation of SPIRIT’s image capability

Using simulation, we compared the data acquisition with the full measurement matrix and the partially filled ones implemented in the conventional linear scan (with bucket signals shown in the left panel of Supplementary Fig. 5e) and SPIRIT. The left image of Supplementary Fig. 6a depicts the full encoding stripe (containing $m = 143$ encoding patterns and four run-way patterns) produced by the procedure described in Supplementary Note 6. The middle image of Supplementary Fig. 6a shows the encoding stripe from the conventional linear scan (see Supplementary Fig. 5d and Supplementary Movie 1). Finally, the encoding stripe used in SPIRIT’s mask plate (containing $m = 91$ encoding patterns and four run-way patterns) is shown in the right image of Supplementary Fig. 6a. The full encoding stripe is 54.7% longer compared to the ones used for the conventional linear scan and SPIRIT. In the encoding stripe of the conventional linear scan, the two regions in the FOV do not have the same orientations as those in SPIRIT. The data acquisition, using the grayscale objects shown in Supplementary Fig. 6b, was simulated with all three encoding schemes. For the case of the conventional linear scan, the orientation difference between the encoding patterns and the objects was ignored in the Hadamard product.

Supplementary Fig. 6c illustrates the reconstruction results obtained using full sampling, the conventional linear scan, and SPIRIT. Due to non-uniform sampling (see the left panel in Supplementary Fig. 5e), the conventional linear scan fails to achieve successful reconstruction. In contrast, SPIRIT’s uniform sampling allows for high-quality reconstruction. The mean intensity profiles along the direction perpendicular to the intensity gradient are shown in Supplementary Fig. 6d. Compared to the result using the full measurement matrix (i.e., the ground truth), the SPIRIT reconstructed image has a relative root-mean-square error of 1.17%. These results thus verify the feasibility of SPIRIT’s data interpolation and reconstruction.

Supplementary Note 8: Details of experimental operation in SPIRIT

All experimental procedures conducted in this work were undertaken indoors, maintaining an ambient temperature and relative humidity of approximately 22 °C and 40% respectively, thereby complying with the environmental considerations stipulated in IEC80601-2-59 (2023) [3] for infrared inspection. To further refine the testing environment, curtains were deployed to encircle the experimental setup, reducing airflow from ventilation and thus minimizing any additional cooling or heating. Moreover, a spray of white paint with high thermal radiation reflectivity was applied to the surfaces of system elements, which mitigated interference from additional infrared radiation sources surrounding the experimental setup, such as incandescent and halogen lamps.

We made off-line invitations on campus with a questionnaire, including questions on gender and glasses-wearing. We recruited 39 human volunteers, including 19 females and 20 males as determined by self-reported gender, to participate in the experiments. The authors informed the selected candidates in person, by email, and/or by telephone. Written informed consent was obtained from every volunteer. Each volunteer received a CA$15 gift card upon completion of the participation.

For inner canthi temperature measurement by SPIRIT, volunteers were asked to remove any eye makeup that might alter the thermal properties of the inner canthi. Furthermore, volunteers were advised to close their eyes for a brief rest before testing and to keep their eyes open during the approximately 15-second data acquisition period. As an example, Supplementary Fig. 7 shows the ground truth images of the four volunteers captured using the thermal camera. The eye contours were extracted and used in Fig. 4a. Information of the volunteers shown in Figs. 4 and 5 in Main Text is listed in Supplementary Table 3.

Supplementary Note 9: Details of statistical analyses for temperature mapping of human inner canthi using SPIRIT

Diurnal variation of temperature

Based on SPIRIT's reconstruction results, a statistical analysis was conducted to assess the significance of temperature differences between adjacent time windows. To account for the temperature variations related to gender and glasses-wearing, we performed the analysis across four groups: males without glasses, males with glasses, females without glasses, and females with

glasses (Supplementary Fig. 8). Each group had seven volunteers. The temperatures of the left inner canthus and the right inner canthus were averaged for each volunteer on each measurement time window. Given the four consecutive measurement time windows, comparisons were conducted three times within each group (i.e., 9:00–11:00 versus 11:00–13:00, 11:00–13:00 versus 13:00–15:00, and 13:00–15:00 versus 15:00–17:00). The number of data points (n_s) used to calculate the inner canthi temperature for each measurement from the 28 volunteers is listed in Supplementary Table 5.

To ensure the robustness of the analysis, the normality of the data distribution within each group was assessed using the Kolmogorov-Smirnov test [4]. The results confirmed that all subsets followed a normal distribution. Then, a two-sample t-test [5,6] was conducted with the null hypothesis assuming no significant temperature difference between adjacent time windows. Moreover, a one-sided (right-tailed) t-test [7] was performed to examine whether temperature differences followed a specific directional trend. Specifically, the second null hypothesis for each comparison stated that the temperature measured between 9:00–11:00 is higher than that between 11:00–13:00, the temperature between 13:00–15:00 is higher than that between 11:00–13:00, and the temperature between 13:00–15:00 is higher than that between 15:00–17:00.

The result is summarized in Supplementary Table 6. For all four groups, the p-values for all three comparisons are below 0.05, sufficient to reject all null hypotheses [8]. These results confirm the statistical significance of the diurnal temperature variation in volunteers.

Gender-related temperature difference

Following the same approach, we examined the significance of the temperature differences between female and male volunteers. The analysis was performed on eight groups. In particular, SPIRIT reconstructions of seven female and seven male volunteers who do not wear glasses were compared for each time window (Supplementary Fig. 9a). Data from seven female and seven male volunteers who wear glasses daily were analyzed in the same manner (Supplementary Fig. 9b).

The statistical tests followed the same method as the diurnal variation analysis. The two-sample t-test was conducted with the null hypothesis of no significant difference in temperatures between male and female volunteers. The one-sided (right-tailed) t-test was performed with the null hypothesis of male volunteers having higher temperatures than female volunteers. As summarized in Supplementary Fig. 9c, the calculated p-values for all comparisons are less than

0.05. Consequently, both null hypotheses are rejected. These results show that the observed temperature differences are statistically significant, confirming that female volunteers exhibit consistently higher temperatures than male volunteers.

Glasses-wearing-related temperature difference

By using the same approach, statistical analysis was conducted to evaluate the significance of temperature differences between volunteers who wear glasses and those who do not (Supplementary Figs. 10a and b). The analysis was performed across specific time windows to account for diurnal variation. The statistical tests followed the same method as the gender-based analysis, with the null hypotheses adapted for this comparison: the first assumed no significant temperature difference between the two groups, while the second specifically tested whether participants who wore glasses had higher temperatures than those who did not. As illustrated in Supplementary Fig. 10c, the p-values for all comparisons are below 0.05, sufficient to reject both null hypotheses. These results establish the statistical significance of the temperature differences, indicating that volunteers who do not wear glasses show higher temperatures compared to those who wear glasses daily.

Supplementary References

1 Sloane, N. J. & Harwit, M. Hadamard Transform Optics, Appendix 203-210, *Academic Press* (1979).
2 Ring, E. & Ammer, K. Infrared thermal imaging in medicine. *Physiological Measurement* **33**, R33 (2012).
3 Medical electrical equipment — Part 2-59: Particular requirements for the basic safety and essential performance of screening thermographs for human febrile temperature screening — Amendment 1. *International Electrotechnical Commission* **IEC 80601-2-59:2017/Amd 1:2023** (2023).
4 Yap, Bee Wah, and Chiaw Hock Sim. Comparisons of various types of normality tests." *Journal of Statistical Computation and Simulation* **81**, 2141-2155 (2011).
5 Kim, Tae Kyun. T test as a parametric statistic. *Korean Journal of Anesthesiology* **68**, 540-546 (2015).
6 Semenick, Doug. Tests and measurements: The T-test. *Strength & Conditioning Journal* **12**, 36-37 (1990).
7 Murphy R. On the use of one-sided statistical tests in biomedical research. *Clinical and Experimental Pharmacology and Physiology* **45**, 109-144 (2018).
8 Sawka, Michael N., C. Bruce Wenger, and Kent B. Pandolf. Thermoregulatory responses to acute exercise - heat stress and heat acclimation. *Comprehensive Physiology* **14**, 157-185 (2010).

Supplementary Figures

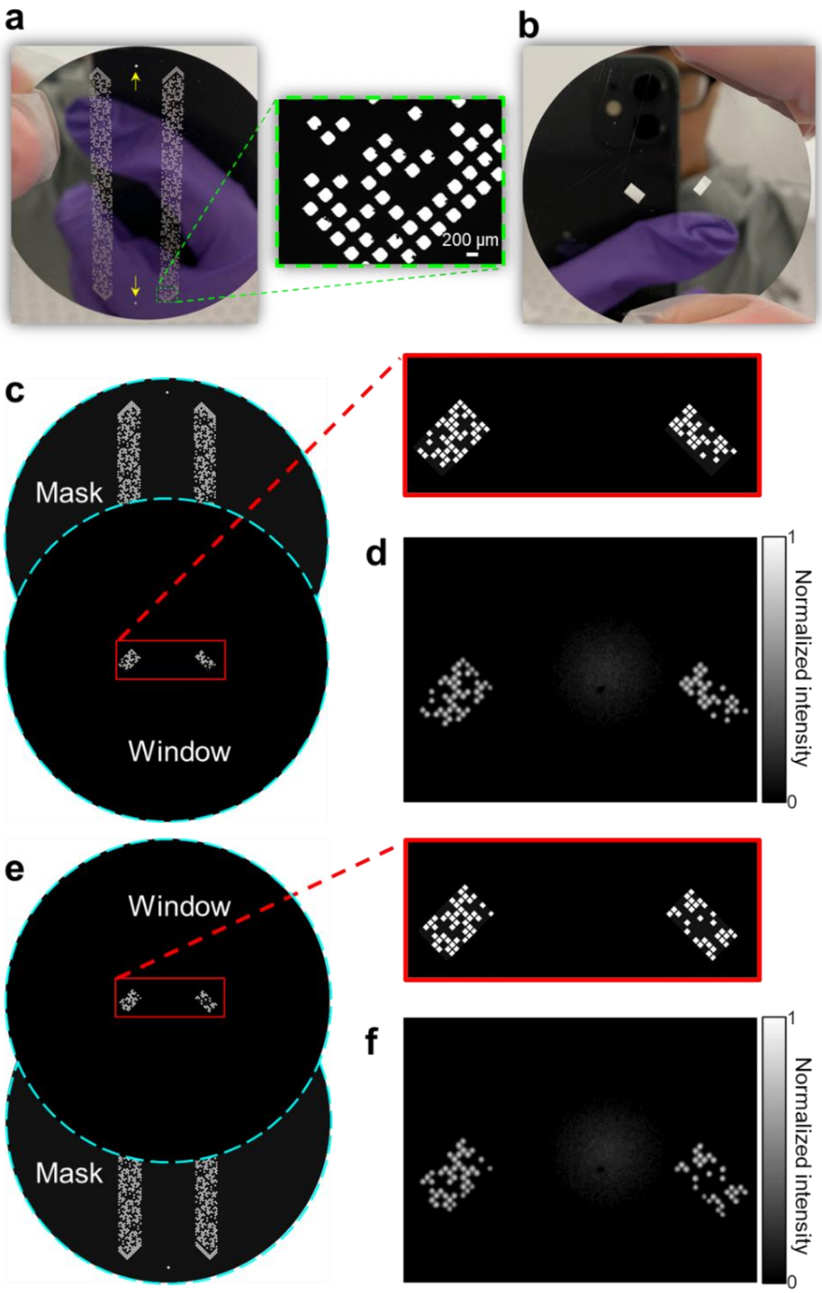

Supplementary Fig. 1. Alignment of the mask plate and the window plate. a Photograph of the mask plate. A close-up view of a microscope-inspected image is shown in the green dashed box. The vertical alignment markers on the mask plate are indicated by the yellow arrows. **b** Photograph of the window plate. **c-f** Two representative images showing the examination of alignment. **c** and **e:** Represented encoding masks, with close-up views shown in the red boxes. **d** and **f**: Images of the encoding masks in (**c**) and (**e**) captured by a thermal camera.

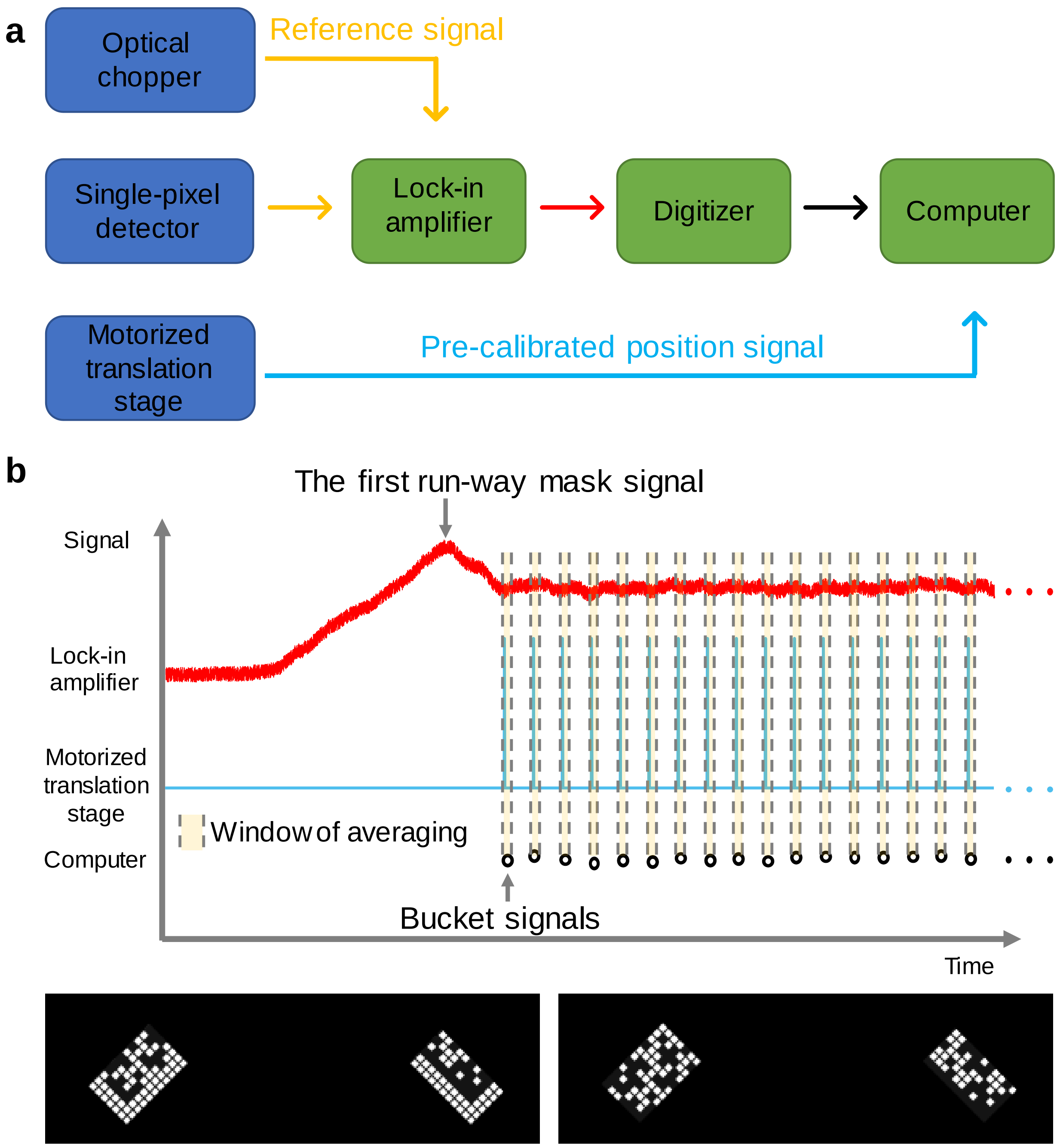

318
319 **Supplementary Fig. 2. Synchronization and data registration in SPIRIT. a** Block diagram
320 illustrating the paths of signals involved in timing and acquisition. **b** Schematic timing diagram
321 illustrating synchronization and data registration. Insets: the first run-way mask (left) and the first
322 encoding mask (right).

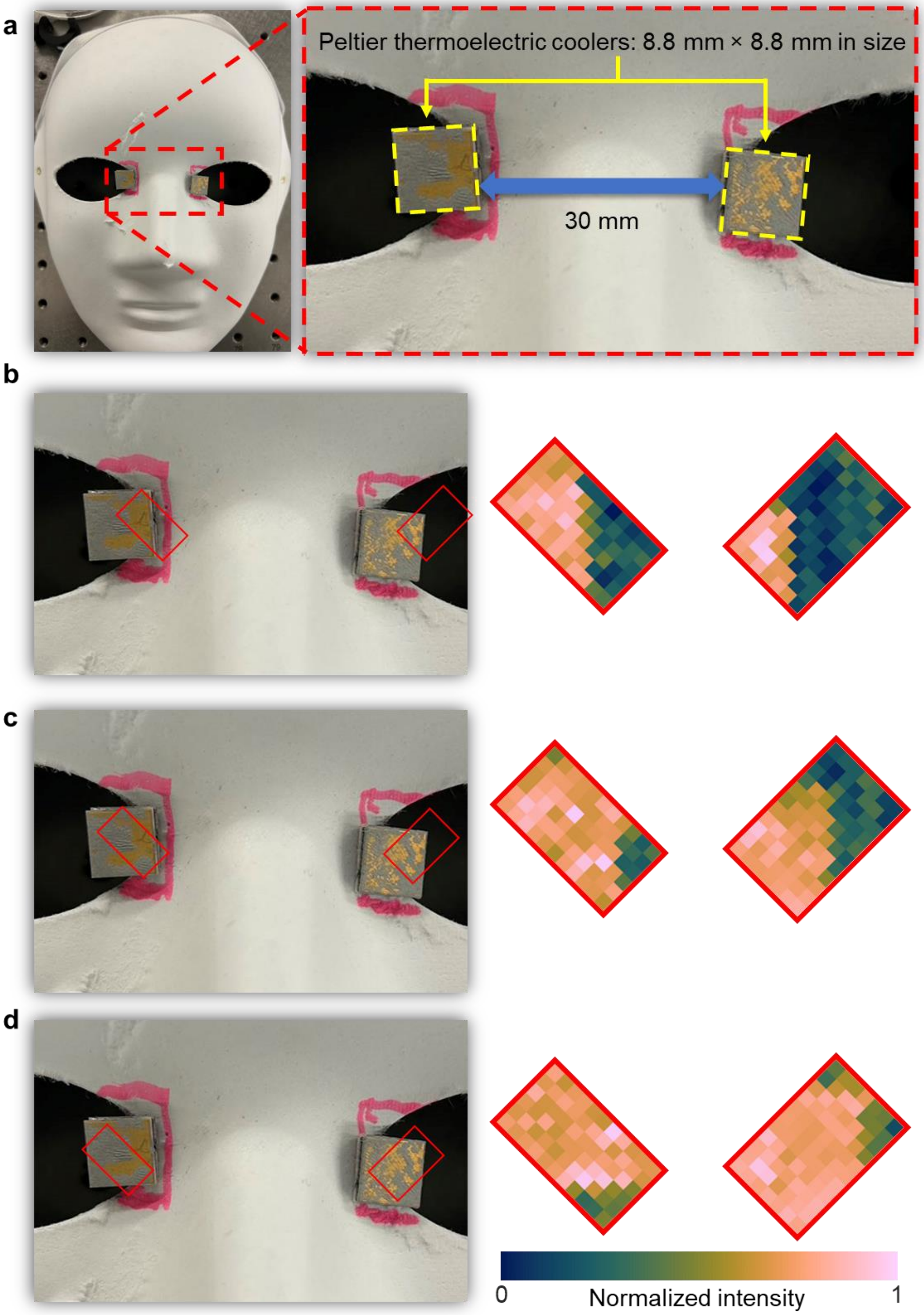

Supplementary Fig. 3. Field-of-view co-registration in SPIRIT. a Photograph of a face mask complemented by two Peltier thermoelectric coolers. Red dashed box: Close-up view of inner-canthus areas. **b–d** Co-registration procedure between the images captured by the visible camera (left row) and the SPIRIT reconstructions (right row).

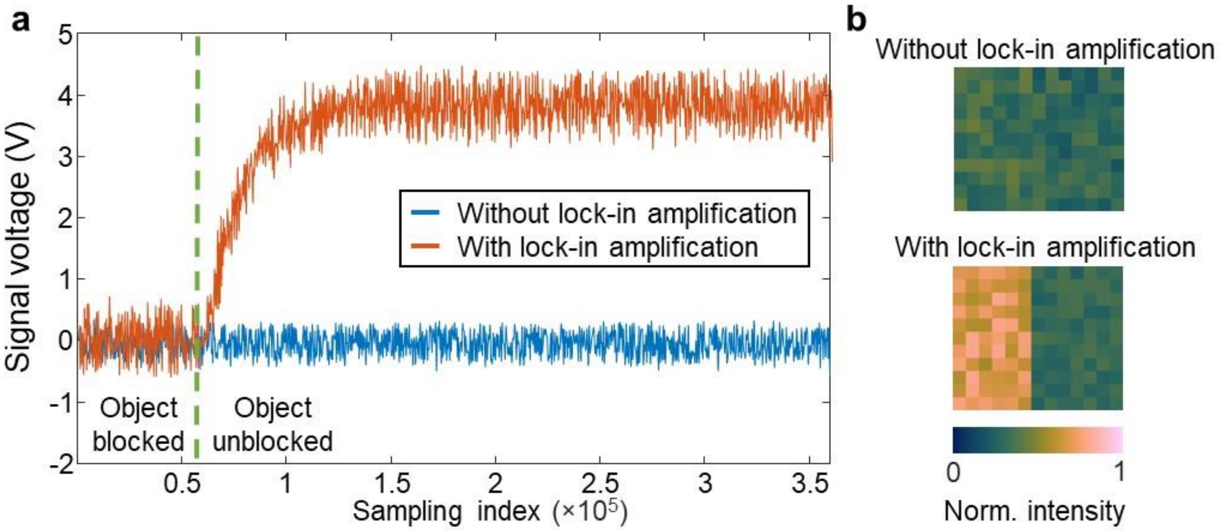

Supplementary Fig. 4. Lock-in amplification for SPIRIT. a Signals acquired while the imaging system is blocked and unblocked. **b** Reconstructions of a half-block feature (i.e., left half open and right half blocked).

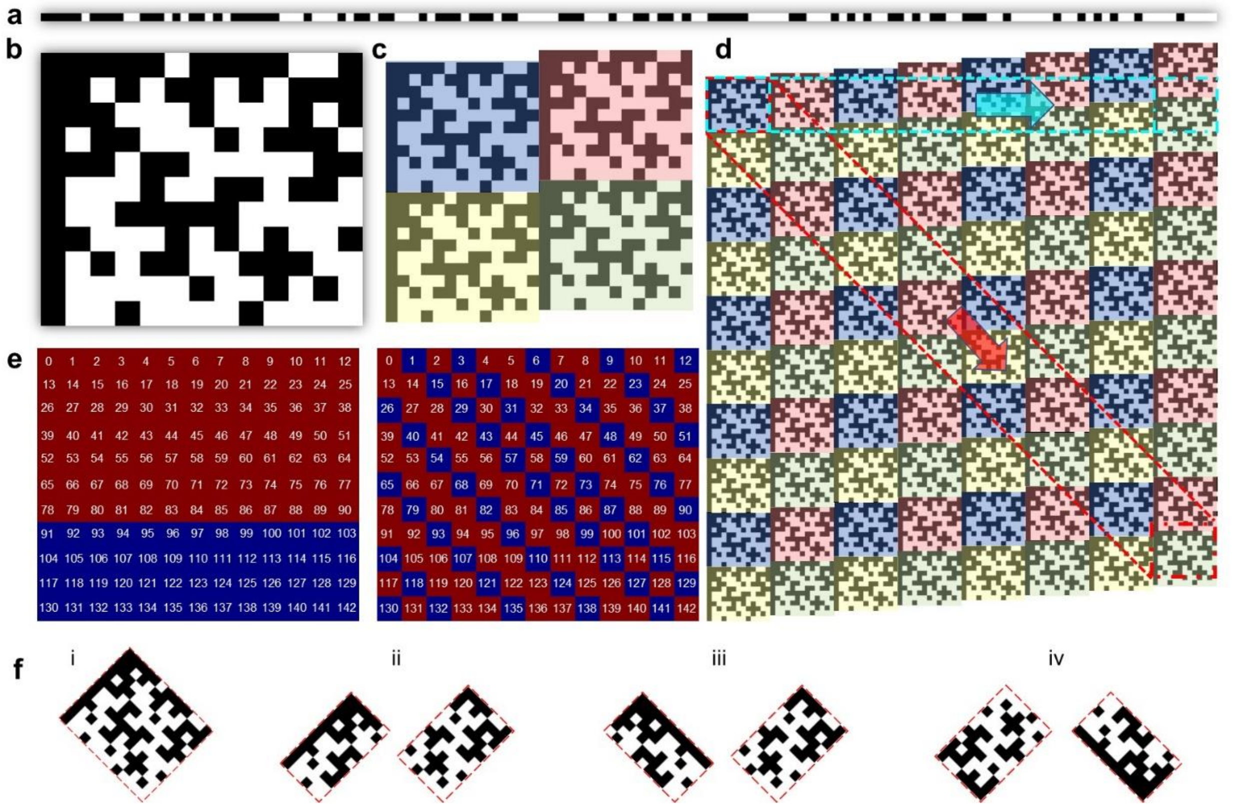

Supplementary Fig. 5. Generation of the encoding patterns for SPIRIT. a First row in the measurement matrix used in SPIRIT. **b** Encoding pattern generated by 2D reshaping of (**a**). **c** Patterns composed of four tiles of (**b**). Shaded colors are used for display purposes. **d** Extended pattern by further duplicating and tiling of (**c**). The red dashed box in the top-left corner shows the first encoding pattern. The red arrow indicates the scan direction. The first encoding pattern and the scan direction of the conventional linear scan are illustrated by the cyan dashed box and the cyan arrow. **e** Comparison of 2D-reshaped bucket signals for conventional linear scan (left) and SPIRIT (right) with the same number of scan steps. Red and blue blocks indicate measured signals and non-measured signals corresponding encoding pattern index. **f** Separation and rearrangement of the first encoding pattern.

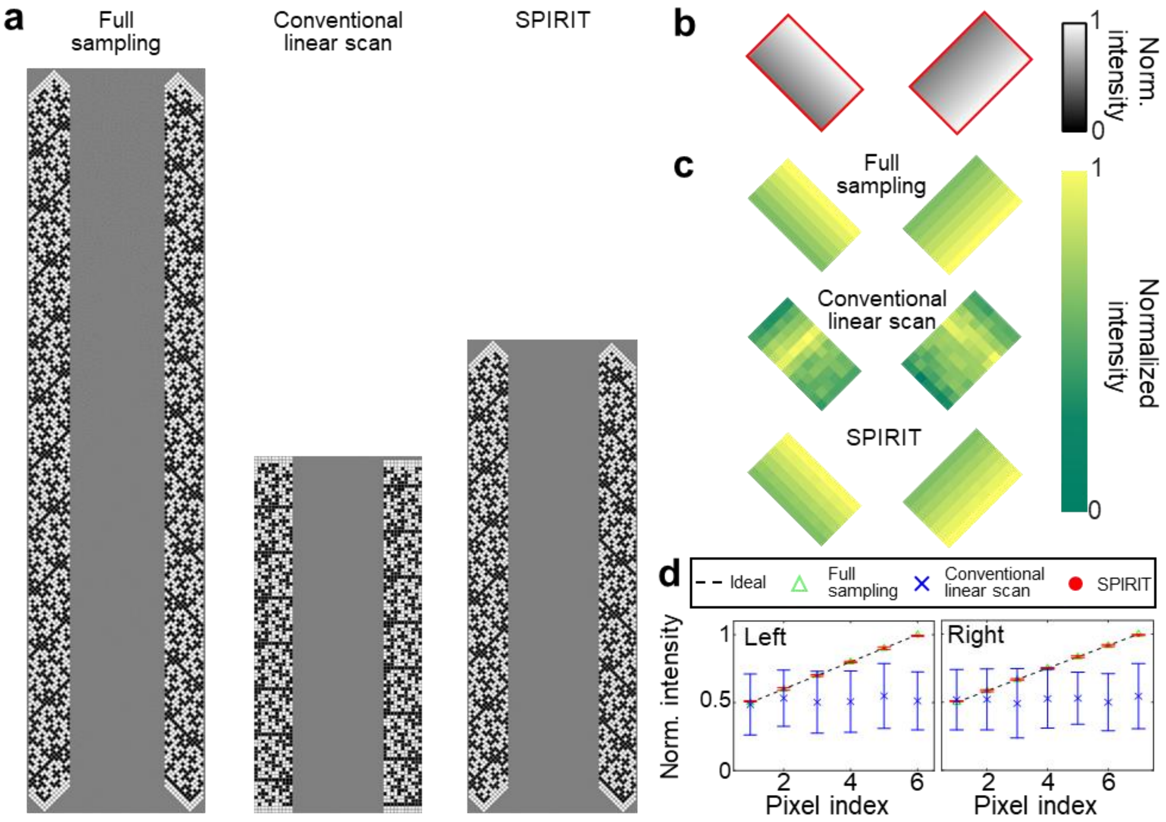

Supplementary Fig. 6. Simulation of SPIRIT. a Illustration of aggregated encoding stripes produced from the full sampling (left), for the conventional linear scan (middle), and for SPIRIT (right). **b** Objects used in simulation. **c** Images reconstructed by using the full sampling (top), conventional linear scan (middle), and SPIRIT (bottom). **d** Mean intensity profiles of full sampling, conventional linear scan, and SPIRIT from (**c**) along the direction perpendicular to the intensity gradient, with $n_s = 11$ for each profile.

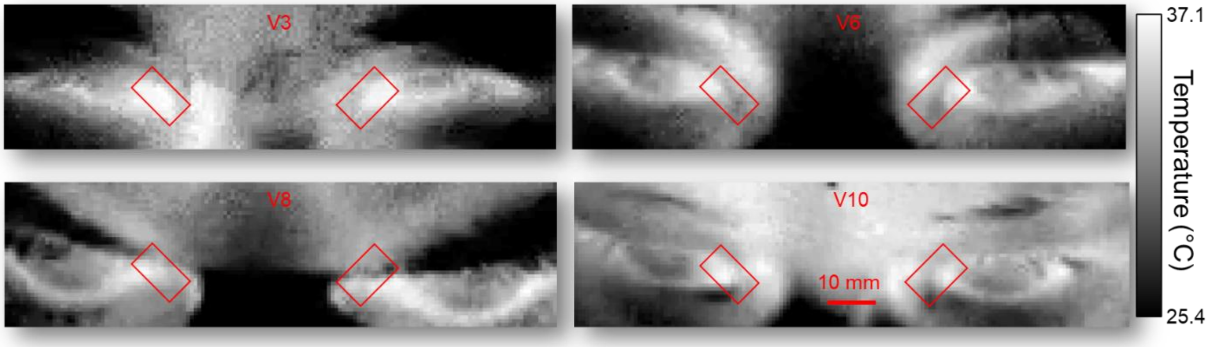

Supplementary Fig. 7. Representative ground-truth images captured by the thermal camera. Red boxes indicate SPIRIT's FOV.

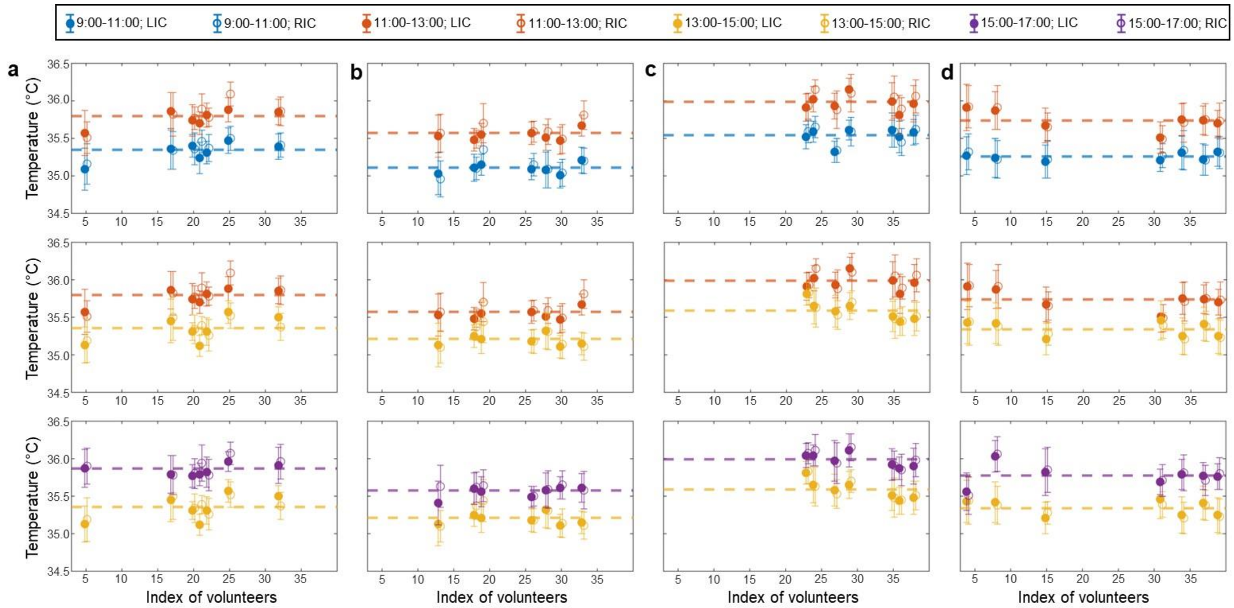

Supplementary Fig. 8. Statistical analysis of diurnal temperature variation of inner canthi. **a** Measurements from male volunteers who do not wear glasses (NWG). From top to bottom: comparisons of 9:00–11:00 versus 11:00–13:00, 11:00–13:00 versus 13:00–15:00, and 13:00–15:00 versus 15:00–17:00. Blue, orange, yellow, and purple dashed lines represent the mean temperatures for each respective time window. Error bars: standard deviation. **b** As (a), but for male volunteers who wear glasses (WG). **c**–**d** As (a) and (b), but for female volunteers in the NWG and WG groups, respectively.

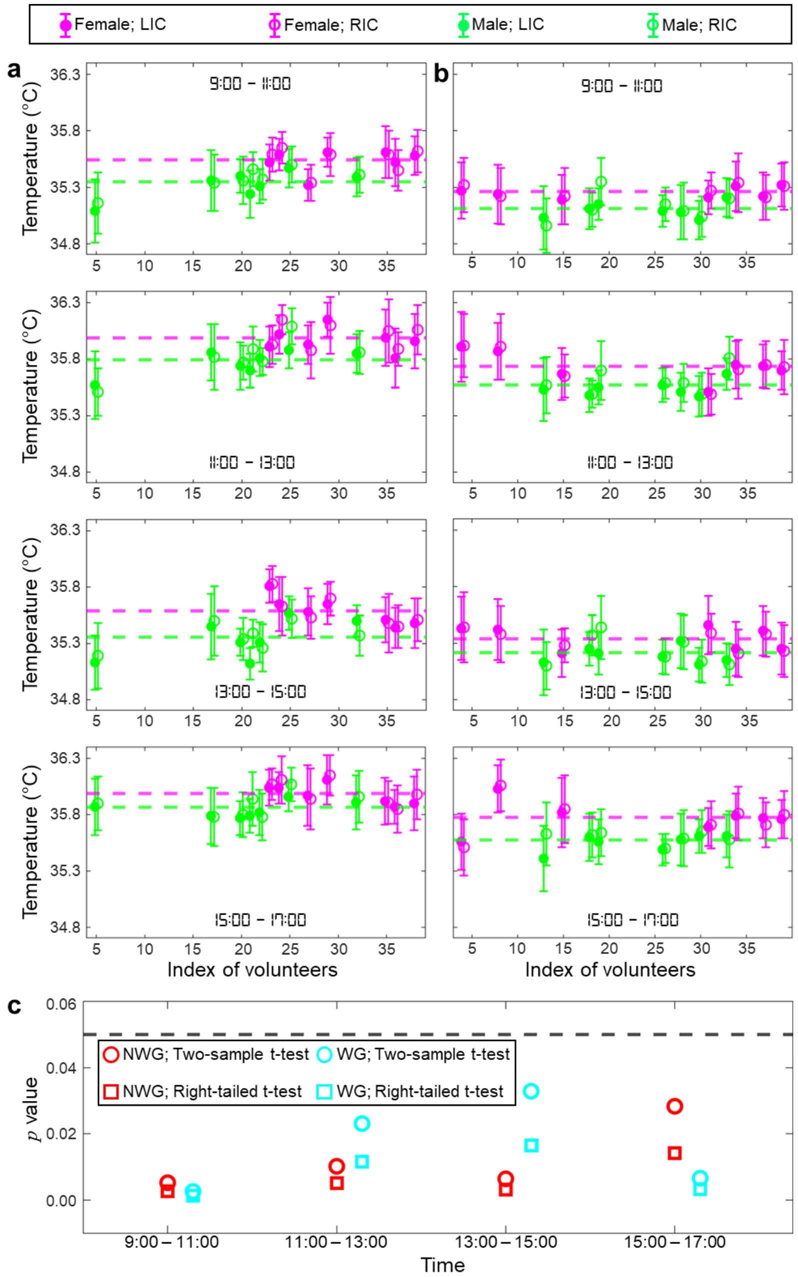

Supplementary Fig. 9. Statistical analysis of temperature differences of inner canthi between female and male volunteers. a Measurements from volunteers who do not wear glasses (NWG) across the four time windows (i.e., 9:00–11:00, 11:00–13:00, 13:00–15:00, and 15:00–17:00). Magenta and green dashed lines mark mean temperatures for the female and male groups, respectively. Error bars: standard deviation. **b** As (a), but for volunteers who wear glasses (WG). **c** Results of the two-sample t-test and the right-tailed t-test. The dashed horizontal line at p=0.05 is the threshold for rejecting the null hypotheses. p<0.05 indicates statistical significance.

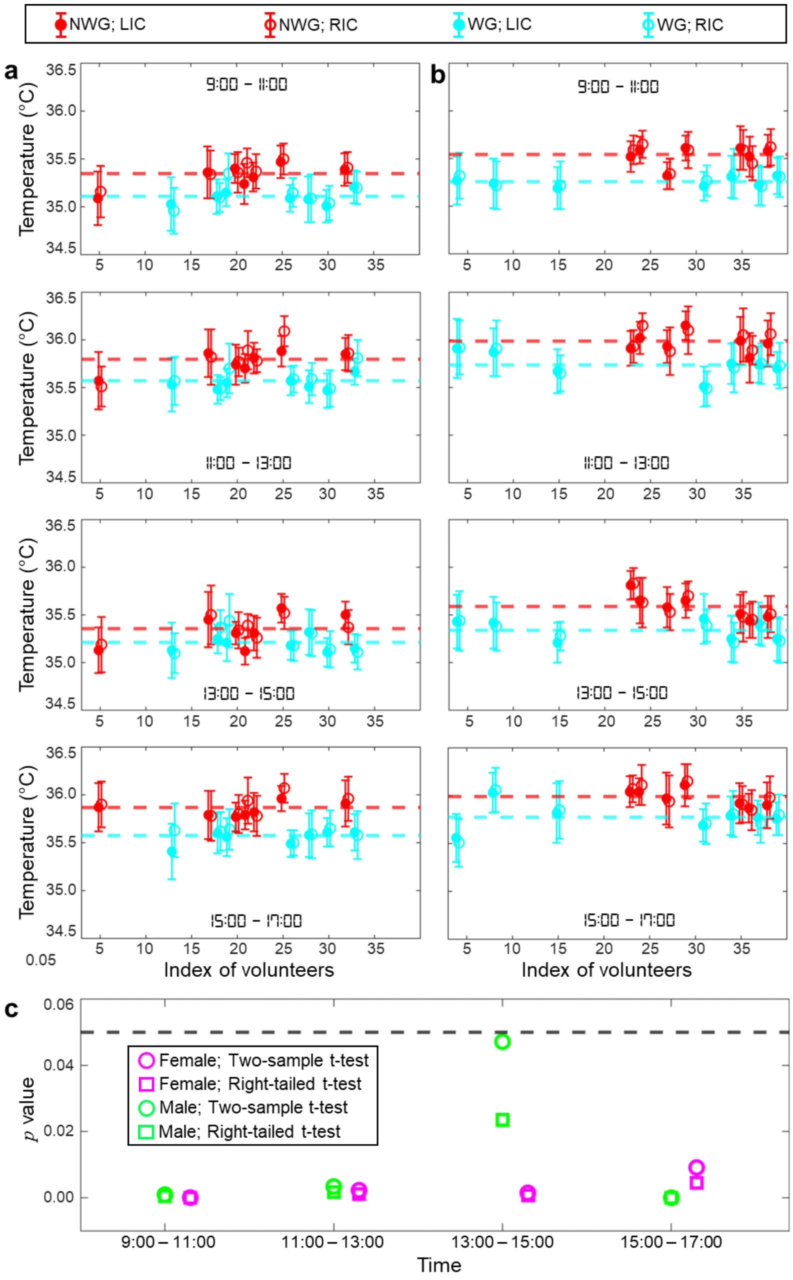

Supplementary Fig. 10. Statistical analysis of temperature differences of inner canthi related to glasses wearing. a Measurements from male volunteers who do not wear glasses (NWG) and wear glasses (WG) across the four time windows (i.e., 9:00–11:00, 11:00–13:00, 13:00–15:00, and 15:00–17:00). Red and cyan dashed lines mark mean temperatures for the NWG and WG groups, respectively. Error bars: standard deviation. **b** As (a), but for female volunteers. **c** Results of the two-sample t-test and the right-tailed t-test. The dashed horizontal line at p=0.05 is the threshold for rejecting the null hypotheses. p<0.05 indicates statistical significance.

Supplementary Tables

n	$Form$	a	b	m	m/n (%)
35	(iii)	5	7	35	100.0
				21	60.0
				14	40.0
35	(iii)	7	5	35	100.0
				30	85.7
63	(ii)	7	9	63	100.0
				45	71.4
				27	42.9
				18	28.6
63	(ii)	9	7	63	100.0
				56	88.9
**143**	**(iii)**	**11**	**13**	143	100.0
				**91**	**63.6**
				52	36.4
143	(iii)	13	11	143	100.0
				132	92.3
255	(ii)	17	15	255	100.0
				240	94.1

Supplementary Table 1. Summary of design-search data for the construction of the encoded stripes used in SPIRIT based on cyclic S-matrices. Bold values indicate the parameters chosen for actual fabrication.

Slit orientation	*Region in the field of view*	*Pixel index*	n_s
0° and 90°	Left	1, 16	1
		2, 15	2
		3, 14	3
		4, 13	4
		5, 12	5
		6 – 11	6
	Right	1, 17	1
		2, 16	2
		3, 15	3
		4, 14	4
		5, 13	5
		6, 12	6
		7 – 11	7
45°	Left	1 – 11	6
	Right	1 – 7	11
-45°	Left	1 – 6	11
	Right	1 – 11	7

Supplementary Table 2. Summary of the number of data points (n_s) used to calculate the mean intensity of each pixel in the line profiles shown in Figs. 3c–f in Main Text.

Index of volunteers	*Gender*	*Wear glasses?*	*Measurement time*
V1	Male	Yes	9:39 *
V2	Male	Yes	9:41 *
V3	Male	Yes	9:47 *
V4	Female	Yes	9:31 ‡
			9:50 *
			11:50 ‡
			13:35 ‡
			15:26 ‡
V5	Male	No	9:42 ‡
			10:04 *
			11:42 ‡
			13:56 ‡
			15:20 ‡
V6	Male	Yes	11:15 *
V7	Male	No	13:20 *
V8	Female	Yes	9:50 ‡
			11:46 ‡
			13:35 *
			13:50 ‡
			15:36 ‡
V9	Female	Yes	13:52 *
V10	Female	Yes	15:05 *
V11	Female	No	15:10 *
V12	Female	No	15:17 *
V13	Male	Yes	9:49 ‡
			11:37 ‡
			13:45 ‡
			15:31 *
			15:41 ‡
V14	Female	Yes	16:01 *
V15	Female	Yes	10:06‡
			12:04*‡
			14:05‡
			15:59‡
V16	Male	Yes	9:58‡
			11:38*‡
			13:46‡
			15:47‡
V17	Male	No	9:46‡
			11:39*‡
			13:31‡
			15:43‡
V18	Male	Yes	9:56‡

			11:35*‡
			13:43‡
			15:36‡
V19	Male	Yes	9:42‡
			11:59*‡
			13:48‡
			15:50‡
V20	Male	No	9:50‡
			12:04*‡
			14:02‡
			15:58‡
V21	Male	No	9:57‡
			11:42*‡
			13:36‡
			15:41‡
V22	Male	No	10:04‡
			11:47*‡
			13:59‡
			15:34‡
V23	Female	No	9:33‡
			11:30*‡
			13:31‡
			15:18‡
V24	Female	No	9:46‡
			11:41*‡
			13:40‡
			15:05‡
V25	Male	No	10:04‡
			11:56*‡
			14:00‡
			15:50‡
V26	Male	Yes	10:12‡
			12:01*‡
			14:07‡
			15:53‡
V27	Female	No	9:59‡
			11:52*‡
			13:42‡
			15:32‡
V28	Male	Yes	9:55‡
			11:54*‡
			13:35‡
			15:40‡
V29	Female	No	9:50‡
			11:41*‡

			13:52‡
			15:59‡
V30	Male	Yes	9:50‡
			12:12*‡
			13:54‡
			15:27‡
V31	Female	Yes	9:35‡
			11:33*‡
			13:30‡
			15:32‡
V32	Male	No	9:40‡
			11:32*‡
			13:55‡
			15:22‡
V33	Male	Yes	9:47‡
			12:11*‡
			14:07‡
			15:50‡
V34	Female	Yes	10:12‡
			11:29*‡
			13:54‡
			15:37‡
V35	Female	No	10:18‡
			11:30*‡
			13:49‡
			15:40‡
V36	Female	No	10:22‡
			11:32*‡
			13:55‡
			15:42‡
V37	Female	Yes	10:00‡
			11:57*‡
			13:34‡
			15:37‡
V38	Female	No	9:44‡
			11:37*‡
			13:40‡
			15:27‡
V39	Female	Yes	9:39‡
			11:40*‡
			13:42‡
			15:43‡

Supplementary Table 3. Information on volunteers. * and ‡ refer to the information used in the analyses presented in Fig. 4 and Fig. 5 in Main Text, respectively.

Index of volunteers	n_s	
	Left inner canthus	*Right inner canthus*
V1	30	35
V2	39	33
V3	42	48
V4	41	22
V5	38	41
V6	21	29
V7	41	35
V8	33	44
V9	42	30
V10	41	45
V11	28	37
V12	37	28
V13	41	39
V14	23	32
V15	29	38
V16	37	22
V17	22	33
V18	23	35
V19	32	28
V20	32	36
V21	32	42
V22	28	40
V23	25	41
V24	35	40
V25	26	27

V26	25	30
V27	42	24
V28	39	33
V29	40	32
V30	31	33
V31	26	23
V32	37	31
V33	42	32
V34	26	31
V35	25	39
V36	23	37
V37	29	40
V38	22	30
V39	31	29

Supplementary Table 4. Summary of n_s used to calculate the inner canthi temperature of the volunteers shown in Fig. 4b in Main text.

Index of volunteers	*Measurement time*	n_s	
		Left inner canthus	*Right inner canthus*
V4	9:31	34	28
	11:50	30	22
	13:35	30	28
	15:26	22	41
V5	9:42	41	28
	11:42	33	32
	13:56	33	39
	15:20	32	41
V8	9:50	33	38
	11:46	41	42
	13:50	35	36
	15:36	24	30
V13	9:49	35	37
	11:37	29	28
	13:45	31	33
	15:41	35	30
V15	10:06	33	30
	12:04	29	38
	14:05	29	25
	15:59	24	37
V16	9:58	26	38
	11:38	37	22
	13:46	37	38
	15:47	28	31
V17	9:46	35	36
	11:39	22	33
	13:31	31	23
	15:43	25	23
V18	9:56	30	35
	11:35	23	35
	13:43	31	39
	15:36	30	31
V19	9:42	23	24
	11:59	32	28
	13:48	33	36
	15:50	34	42
V20	9:50	41	25
	12:04	32	36
	14:02	32	25
	15:58	37	26
V21	9:57	40	28
	11:42	32	42

	13:36	34	35
	15:41	34	35
V22	10:04	40	23
	11:47	28	40
	13:59	42	24
	15:34	29	38
V23	9:33	35	40
	11:30	25	41
	13:31	42	42
	15:18	26	28
V24	9:46	30	42
	11:41	35	40
	13:40	29	28
	15:05	25	27
V25	10:04	42	42
	11:56	26	27
	14:00	36	27
	15:50	28	23
V26	10:12	26	24
	12:01	25	30
	14:07	29	25
	15:53	34	38
V27	9:59	28	33
	11:52	42	24
	13:42	28	38
	15:32	41	38
V28	9:55	41	35
	11:54	39	33
	13:35	22	42
	15:40	36	22
V29	9:50	22	25
	11:41	40	32
	13:52	38	33
	15:59	26	40
V30	9:50	35	41
	12:12	31	33
	13:54	24	30
	15:27	23	29
V31	9:35	26	37
	11:33	26	23
	13:30	37	32
	15:32	29	39
V32	9:40	41	30
	11:32	37	31
	13:55	28	29

	15:22	30	31
V33	9:47	25	22
	12:11	42	32
	14:07	33	34
	15:50	34	35
V34	10:12	26	30
	11:29	26	31
	13:54	29	25
	15:37	33	40
V35	10:18	40	33
	11:30	25	39
	13:49	26	33
	15:40	24	34
V36	10:22	38	26
	11:32	23	37
	13:55	38	23
	15:42	36	35
V37	10:00	33	39
	11:57	29	40
	13:34	23	24
	15:37	37	24
V38	9:44	30	24
	11:37	22	30
	13:40	29	29
	15:27	35	30
V39	9:39	31	41
	11:40	31	29
	13:42	33	40
	15:43	26	35

Supplementary Table 5. Summary of n_s used to calculate the inner canthi temperature of the volunteers for the statistical analysis shown in Supplementary Figs. 8–10.

Time window	*Male*				*Female*			
	Not wear glasses		*Wear glasses*		*Not wear glasses*		*Wear glasses*	
	Two-sample t-test	*Right-tailed t-test*	*Two-sample t-test*	*Right-tailed t-test*	*Two-sample t-test*	*Right-tailed t-test*	*Two-sample t-test*	*Right-tailed t-test*
9:00–11:00 versus 11:00–13:00	1.8×10^{-5}	9.1×10^{-6}	5.9×10^{-7}	3.0×10^{-7}	3.0×10^{-6}	1.5×10^{-6}	2.1×10^{-6}	1.0×10^{-6}
11:00–13:00 versus 13:00–15:00	5.3×10^{-5}	2.7×10^{-5}	1.2×10^{-5}	5.9×10^{-6}	3.4×10^{-5}	1.7×10^{-5}	4.4×10^{-5}	2.2×10^{-5}
13:00–15:00 versus 15:00–17:00	2.2×10^{-6}	1.1×10^{-6}	1.5×10^{-6}	7.7×10^{-7}	3.0×10^{-5}	1.5×10^{-5}	3.3×10^{-5}	1.7×10^{-5}

Supplementary Table 6. p-values of the t-test for the diurnal variation in human temperature. For each t-test, n_s = 7.
