# Supplementary material for: Single-pixel infrared imaging thermometry maps human inner canthi temperature
Source: Nat Commun. 2025 Oct 6;16:8885. doi: 10.1038/s41467-025-64125-3 (PMC12501256; doi:10.1038/s41467-025-64125-3)
Supplement: Supplementary file 2 — Description of Additional Supplementary Files [file 41467_2025_64125_MOESM2_ESM.pdf]

Description of Additional Supplementary Files

Supplementary Movie 1: Animated illustration of SPIRIT’s working principle.
