# Supplementary material for: Single-pixel infrared imaging thermometry maps human inner canthi temperature
Source: Nat Commun. 2025 Oct 6;16:8885. doi: 10.1038/s41467-025-64125-3 (PMC12501256; doi:10.1038/s41467-025-64125-3)
Supplement: Supplementary file 4 — Reporting summary [file 41467_2025_64125_MOESM4_ESM.pdf]

nature portfolio

Corresponding author(s): Jinyang Liang

Last updated by author(s): Aug 14, 2025

Reporting Summary

Nature Portfolio wishes to improve the reproducibility of the work that we publish. This form provides structure for consistency and transparency in reporting. For further information on Nature Portfolio policies, see our Editorial Policies and the Editorial Policy Checklist.

Statistics

For all statistical analyses, confirm that the following items are present in the figure legend, table legend, main text, or Methods section.

n/a	Confirmed	
☐	☒	The exact sample size (n) for each experimental group/condition, given as a discrete number and unit of measurement
☐	☒	A statement on whether measurements were taken from distinct samples or whether the same sample was measured repeatedly
☐	☒	The statistical test(s) used AND whether they are one- or two-sided<br>*Only common tests should be described solely by name; describe more complex techniques in the Methods section.*
☐	☒	A description of all covariates tested
☐	☒	A description of any assumptions or corrections, such as tests of normality and adjustment for multiple comparisons
☐	☒	A full description of the statistical parameters including central tendency (e.g. means) or other basic estimates (e.g. regression coefficient) AND variation (e.g. standard deviation) or associated estimates of uncertainty (e.g. confidence intervals)
☐	☒	For null hypothesis testing, the test statistic (e.g. F, t, r) with confidence intervals, effect sizes, degrees of freedom and P value noted<br>*Give P values as exact values whenever suitable.*
☒	☐	For Bayesian analysis, information on the choice of priors and Markov chain Monte Carlo settings
☒	☐	For hierarchical and complex designs, identification of the appropriate level for tests and full reporting of outcomes
☒	☐	Estimates of effect sizes (e.g. Cohen's d, Pearson's r), indicating how they were calculated

Our web collection on statistics for biologists contains articles on many of the points above.

Software and code

Policy information about availability of computer code

Data collection	The authors have developed the data collection code by incorporating open source control functions for specific equipment used in the experiments, including the motorized translation stage and the data acquisition card. All control codes are designed to be executed within the MATLAB platform.
Data analysis	The authors have developed the data analysis code based on well-founded interpolation methods and matrix inversion. The MATLAB codes for image reconstruction are provided as the Supplementary Software Package.

For manuscripts utilizing custom algorithms or software that are central to the research but not yet described in published literature, software must be made available to editors and reviewers. We strongly encourage code deposition in a community repository (e.g. GitHub). See the Nature Portfolio guidelines for submitting code & software for further information.

Data

Policy information about availability of data

All manuscripts must include a data availability statement. This statement should provide the following information, where applicable:

- Accession codes, unique identifiers, or web links for publicly available datasets
- A description of any restrictions on data availability
- For clinical datasets or third party data, please ensure that the statement adheres to our policy

All data needed to evaluate the findings of this study are present in the paper and Supplementary Information. Raw data are provided with the Source Data Package (https://doi.org/10.5281/zenodo.16790723).

Research involving human participants, their data, or biological material

Policy information about studies with human participants or human data. See also policy information about sex, gender (identity/presentation), and sexual orientation and race, ethnicity and racism.

Reporting on sex and gender	The authors recruited 39 human volunteers, including 19 females and 20 males as determined by self-reported gender, to participate in the experiments. The gender data, while linked to the experimental results, has been anonymized to ensure confidentiality and is shared only in conjunction with these results. Analyzing the variation in reconstruction outcomes by gender is a principal focus and has been documented in the results submitted for review.
Reporting on race, ethnicity, or other socially relevant groupings	In our study, variations in skin tone were considered non-relevant to the inner canthi temperature. Thus, to avoid unnecessary indirect identifiers to the volunteers, we did not report the information of race, ethnicity or any other socially relevant groupings.
Population characteristics	The authors documented whether the participants were long-term wearer of glasses, based on self-reports. This characteristic was studied to evaluate the impact of glasses wearing on inner canthi temperature.
Recruitment	The authors made off-line invitations on our campus with a questionnaire, including several questions on gender, and glasses-wearing. Candidates were grouped according to the above-mentioned requirements. The authors informed the selected candidates in person, by email, and/or by telephone. Written informed consent was obtained from every participant. Each volunteer received a CA$15 gift card on completion of the participation.
Ethics oversight	Human Ethics Research Committee at the Institut National de la Recherche Scientifique, Université du Québec.

Note that full information on the approval of the study protocol must also be provided in the manuscript.

Field-specific reporting

Please select the one below that is the best fit for your research. If you are not sure, read the appropriate sections before making your selection.

☒ Life sciences ☐ Behavioural & social sciences ☐ Ecological, evolutionary & environmental sciences

For a reference copy of the document with all sections, see nature.com/documents/nr-reporting-summary-flat.pdf

Life sciences study design

All studies must disclose on these points even when the disclosure is negative.

Sample size	In our study, we recruited 39 human volunteers, consisting of 19 females and 20 males, to measure the inner canthi temperature. The sample size was chosen based on the exploratory nature of the study and resource availability. Although no formal sample size calculation was conducted, the equal representation of gender aimed to minimize bias and provide preliminary insights into gender-related differences in inner canthi temperature.
Data exclusions	No data were excluded from the analyses.
Replication	To ensure the reproducibility of our experimental findings, several measures were implemented during the study. We measured the temperature of inner canthi of 39 volunteers at various times throughout the day to assess the consistency of our measurements across different conditions and times. Additionally, to validate the robustness and reliability of our reconstruction algorithm, we performed repeated measurements on a subset of 29 volunteers (the other 10 volunteers provided only one measurement due to personal availability). Each one of these 29 volunteers was measured multiple times over a single day. These repeated measurements allowed us to assess the temporal stability and reliability of the reconstruction algorithm under varying conditions. Finally, by cross-referencing the results with ground truth data from a calibrated thermal camera, our replication attempts confirmed the feasibility of our system for providing consistent and accurate temperature measurements.
Randomization	To assess the impact of gender and glasses-wearing on inner canthi temperature readings, we balanced our study groups by both gender and glasses wearing. Specifically, after excluding one fever-positive volunteer, the remaining 28 fever-negative individuals were evenly divided by gender (14 males and 14 females), with each gender subgroup further split evenly between glasses wearers and non-wearers (7 each).

	Temperature comparisons were then made between these paired subgroups, with measurements taken at approximately the same time to control for diurnal variations. This careful design allowed us to attribute any observed differences directly to the gender or glasses-wearing.
Blinding	In our study, blinding of the investigators to group allocation during data collection and analysis was not implemented. The primary reason for this was the visible nature of the variables under investigation, specifically, gender and glasses-wearing. These characteristics are inherently apparent, making it impossible for investigators to remain unaware of the group allocation while measuring inner canthi temperatures.We recognize that the lack of blinding could potentially introduce observer bias during measurement and analytic bias during data interpretation. However, these risks were mitigated by relying on objective statistical analysis, with temperature data processed through our reconstruction algorithm to standardize data processing and reduce subjective influence. In addition, in line with the guidelines of the Human Ethics Research Committee at the Institut National de la Recherche Scientifique, rigorous measures were implemented to anonymize data and ensure confidentiality at every stage of the study.

Reporting for specific materials, systems and methods

We require information from authors about some types of materials, experimental systems and methods used in many studies. Here, indicate whether each material, system or method listed is relevant to your study. If you are not sure if a list item applies to your research, read the appropriate section before selecting a response.

Materials & experimental systems

n/a	Involved in the study
☒	☐ Antibodies
☒	☐ Eukaryotic cell lines
☒	☐ Palaeontology and archaeology
☒	☐ Animals and other organisms
☒	☐ Clinical data
☒	☐ Dual use research of concern
☒	☐ Plants

Methods

n/a	Involved in the study
☒	☐ ChIP-seq
☒	☐ Flow cytometry
☒	☐ MRI-based neuroimaging

Plants

Seed stocks	N/A
Novel plant genotypes	N/A
Authentication	N/A
